# The cognitive and affective pathways of FinTech adoption: a trait-to-state-to-intention mechanism

**DOI:** 10.3389/fpsyg.2026.1873926

**Published:** 2026-08-24

**Authors:** Ling Li, Cong Zhao

**Affiliations:** School of Economics and Management, Shangqiu Institute of Technology, Shangqiu, China

**Keywords:** consumer psychology, FinTech adoption, TAM, technology readiness, trust

## Abstract

**Introduction:**

This study investigates how consumers’ technology readiness traits shape key psychological drivers—trust, perceived usefulness (PU), and perceived ease of use (PEOU)—and how these factors jointly determine FinTech adoption intention.

**Methods:**

Survey data from 336 Chinese consumers were analyzed using Partial Least Squares Structural Equation Modeling (PLS-SEM) to test an integrated Technology Acceptance Model (TAM), trust, and technology readiness framework.

**Results:**

The results reveal that PU and PEOU significantly predict adoption intention, with PEOU also exerting an indirect effect via PU. Trust acts as a foundational enabler, directly increasing adoption intention while simultaneously enhancing both PU and PEOU. Crucially, the dimensions of technology readiness operate through distinct cognitive and affective pathways. Optimism indirectly drives adoption by fostering trust, PU, and PEOU, whereas innovativeness directly increases intention while enhancing trust and PEOU. Conversely, inhibitor traits manifest differently: discomfort specifically reduces PEOU, whereas insecurity directly undermines trust.

**Discussion:**

The findings demonstrate that technology readiness influences FinTech adoption through distinct psychological pathways rather than as a uniform set of antecedents. The study thus provides a trait-to-state-to-intention explanation of how stable consumer predispositions shape FinTech adoption.

## 1 Introduction

The rapid digitization of financial services presents a unique and high-stakes environment for studying human decision-making under uncertainty (Gomber et al., 2018; Thakor, 2020). When interacting with Financial Technology (FinTech), consumers are required to navigate self-directed, intangible digital interfaces that often trigger significant cognitive and behavioral apprehension (Parasuraman and Colby, 2015). Unlike routine digital interactions, financial contexts involve high perceived vulnerability, making them an ideal domain to examine how individual psychological predispositions and transient cognitive states govern technology acceptance (McKnight et al., 2002; Gefen et al., 2003). A central question for cyberpsychology and human-computer interaction is: how do stable personality traits shape the cognitive appraisals and affective responses that ultimately drive behavior in high-risk digital environments (Lin and Hsieh, 2007; Walczuch et al., 2007)?

From a cognitive decision perspective, adoption intention reflects a user’s evaluation of whether a system’s interaction feels manageable and whether its benefits outweigh the psychological risks. The Technology Acceptance Model (TAM) offers a parsimonious explanation of adoption intention, proposing that users’ intentions are primarily driven by perceived usefulness (PU) and perceived ease of use (PEOU) (Davis, 1989). TAM has been repeatedly validated across digital contexts, emphasizing that usefulness is typically a dominant driver of intention while ease of use influences intention both directly and indirectly via usefulness (Venkatesh and Davis, 2000). For marketing decision-making, these TAM beliefs map onto core levers: communicating functional outcomes (PU) and removing friction in the customer journey (PEOU). However, FinTech contexts introduce substantial uncertainty and perceived risk, meaning that cognitive belief formation is not purely instrumental and may depend strongly on the psychological state of trust (Gefen et al., 2003).

Trust is widely treated as a central relational belief under uncertainty and a willingness to be vulnerable (Mayer et al., 1995). It becomes especially salient when outcomes cannot be fully verified at the time of use (McKnight et al., 2002). Prior work integrating trust with TAM argues that adoption intentions depend jointly on perceptions of the technology artifact (PU and PEOU) and trust in the vendor or service context, which is particularly relevant for financial services where perceived losses can be substantial (Gefen et al., 2003; Kim et al., 2009). Psychologically, trust shapes how consumers interpret uncertainty, evaluate system reliability, and process interface interactions. Trust can therefore influence adoption intention directly and indirectly by strengthening PU—the confidence that promised benefits will materialize—and PEOU—the reduced apprehension during interaction (McKnight et al., 2002).

A further gap concerns why consumers differ systematically in how they form these cognitive appraisals and psychological states in the first place. Technology readiness (TR) conceptualizes stable individual differences in the propensity to embrace new technologies through two motivators (optimism and innovativeness) and two inhibitors (discomfort and insecurity) (Parasuraman and Colby, 2015). TR has been used to explain evaluations of self-service technologies (Liljander et al., 2006) and has also been integrated with TAM to argue that trait-like readiness can shape PU and PEOU, providing a theoretically coherent pathway from personal predispositions to adoption intentions (Lin and Hsieh, 2007; Walczuch et al., 2007). In FinTech, where services are self-directed and intangible, TR may be especially consequential because it can bias consumers’ interpretation of uncertainty, their cognitive appraisal of instrumental value, and their felt interaction effort. Treating TR as multidimensional is important for understanding human-technology interaction because each dimension implies different cognitive barriers and distinct behavioral responses (Parasuraman and Colby, 2015).

Although previous FinTech adoption studies have successfully integrated Technology Readiness, Trust, and the Technology Acceptance Model (Lin and Hsieh, 2007; Walczuch et al., 2007; Blut and Wang, 2020), these models remain largely antecedent-oriented. They primarily identify which factors influence adoption intention, while providing comparatively limited explanation of the psychological process through which stable individual predispositions are translated into behavioral intention. Technology Readiness dimensions are typically treated as parallel predictors of technology acceptance, implicitly assuming that their influence follows a common psychological route.

This study argues that such an assumption oversimplifies consumer decision-making in FinTech environments. Adoption of financial technologies requires consumers to simultaneously evaluate functional utility, interaction complexity, institutional reliability, and financial uncertainty. Under these conditions, different personality traits are unlikely to influence behavior through identical psychological mechanisms. Instead, each Technology Readiness dimension may activate a distinct sequence of psychological states before behavioral intention is formed.

Accordingly, this study proposes a trait-to-state-to-intention framework that reconceptualizes Technology Readiness as a differentiated psychological system rather than a homogeneous set of adoption antecedents. The framework explains how stable technology-related personality traits (Trait) selectively shape transient psychological states, including trust, perceived usefulness, and perceived ease of use (State), which subsequently determine FinTech adoption intention (Intention). By shifting the theoretical focus from identifying significant predictors to explaining the psychological transformation from enduring dispositions to behavioral intention, the proposed framework provides a process-based explanation of FinTech adoption that extends existing TR–TAM integration beyond the combination of established constructs. Consequently, the proposed framework contributes at the level of explanatory logic rather than construct composition. Its theoretical value lies in specifying the psychological sequence through which technology-related personality traits become adoption behavior, rather than introducing additional antecedents into existing technology acceptance models.

## 2 Literature review

### 2.1 FinTech in the financial service sector

FinTech is widely treated as an umbrella concept covering technology-enabled innovations across payments, lending/credit, insurance, and investment services, rather than a single, uniform phenomenon (Gomber et al., 2017; Thakor, 2020). This breadth is analytically useful for mapping change across financial services, but it can also weaken construct clarity because studies sometimes conflate “FinTech” with general digitalization or bank IT modernization, making cross-study comparisons difficult (Gomber et al., 2017). Building on an information-systems perspective, the “FinTech revolution” is often framed as a combined process of innovation, disruption, and transformation that reshapes business models and market structure—not merely customer-facing apps (Gomber et al., 2018).

Empirical work in mortgage markets shows that technology-driven lending can reduce operational frictions (e.g., faster processing) without increasing default rates, indicating that some FinTech applications produce real efficiency gains (Fuster et al., 2019). However, the same stream of evidence suggests that efficiency improvements do not automatically imply broad-based financial inclusion, because adoption and allocation outcomes depend on institutional constraints and how digital channels are integrated into underwriting and distribution (Fuster et al., 2019). Critically, research also cautions that observed FinTech growth in some segments may reflect regulatory and institutional incentives—shifting intermediation outside banks—rather than purely superior technology, which complicates “disruption” narratives (Buchak et al., 2018). Recent empirical investigations reinforce that the widespread adoption of digital financial services fundamentally address these market dynamics by mitigating traditional information asymmetries and reducing operational friction. For instance, Chamboko (2024) applied a time-to-event analysis to demonstrate that digital financial services adoption significantly alleviates financial market imperfections, although adoption propensity varies considerably across demographic and socio-economic segments.

Consumers can benefit from FinTech adoption through faster and more efficient service delivery, as evidence from digital mortgage lending shows shorter processing times without higher default rates, implying genuine efficiency gains rather than mere risk shifting (Fuster et al., 2019). FinTech can also expand access to credit, with research indicating that FinTech lenders are more likely to operate in underserved locations and in areas with fewer bank branches, thereby improving availability where traditional channels are limited (Jagtiani and Lemieux, 2018). At a broader welfare level, higher FinTech adoption has been associated with increased financial inclusion and reduced consumption inequality, suggesting potentially beneficial distributional effects for households (Yang and Zhang, 2022). For investors, robo-advisory services may enhance portfolio decisions and risk-adjusted outcomes, particularly by reducing behavioral frictions and enabling scalable advice delivery (Back et al., 2023; Liu et al., 2023). From the institutional perspective, studies further link FinTech adoption and innovation to improvements in bank efficiency and performance outcomes, especially when digital innovation complements banks’ capabilities and strategic execution (Lee et al., 2021; Wang et al., 2021).

### 2.2 Technology acceptance model

Within cyberpsychology, TAM is fundamentally a cognitive evaluation model. It posits that human-computer interaction is governed by an individual’s subjective cognitive construction of an artifact’s performance gains (PU) and the psychological effort required to operate it (PEOU) (Davis, 1989). TAM explains technology adoption intention through two key beliefs: PU and PEOU. PU is defined as the extent to which using a system enhances an individual’s performance, while PEOU reflects the degree to which using the system is free of effort (Davis, 1989). Empirical tests of TAM show that both PU and PEOU predict behavioral intention and use, with PU typically exerting the stronger direct effect and PEOU influencing intention both directly and indirectly via PU (Davis, 1989; Venkatesh and Davis, 2000). Subsequent research confirms TAM’s robustness and parsimony across diverse digital contexts, including online banking, mobile payments, and mobile financial services, making it a central framework for understanding FinTech adoption.

In FinTech and mobile financial service settings, PU consistently emerges as a primary driver of users’ adoption intention such as mobile payments, mobile banking, and other digital financial platforms. When consumers believe that FinTech services enhance transaction efficiency, convenience, or financial management outcomes, they are more willing to adopt and continue using these services (e.g., Daragmeh et al., 2021; Tsai et al., 2022; Tian and Chan, 2024). PEOU also plays a critical role, as interfaces that are perceived as simple and intuitive reduce cognitive and operational effort, which in turn increases both perceived usefulness and adoption intention for mobile and digital financial services (Davis, 1989; Daragmeh et al., 2021). Studies focusing on digital banking and mobile payments further report that easier-to-use platforms are viewed as more beneficial and trustworthy, which supports the mediating role of PU in the PEOU to intention link in financial contexts (Albuainain and Ashby, 2025; Amnas et al., 2025). Current empirical evidence strongly corroborates these foundational TAM mechanisms. Recent research confirms that perceived usefulness remains one of the strongest predictors of behavioral intention to use FinTech, emphasizing that evident economic benefits and intuitive design consistently incentivize user adoption in emerging markets (Vardari and Hameli, 2025).

Given that FinTech solutions (e.g., investment platforms, digital wallets, mobile banking apps) are accessed through user interfaces and self-service processes, the core TAM relationships are especially salient. Studies of TAM in mobile financial services show that PU and PEOU remain among the most consistent and significant predictors of adoption intention across countries, technologies, and study designs, reinforcing their relevance for explaining consumers’ FinTech adoption intentions (Zhou, 2011; Shaikh and Karjaluoto, 2015). Furthermore, the original TAM proposition that PEOU positively influences PU is repeatedly supported in financial-service applications, where users who perceive FinTech services as easy to operate are more likely to regard them as useful for everyday financial tasks.

Building on the original TAM and its applications in financial services, the following hypothesis is posited:

*H1*: PU is positively associated with a consumer’s FinTech adoption intention.

*H2*: PEOU is positively associated with a consumer’s FinTech adoption intention.

*H3*: PEOU is positively associated with a consumer’s perceived usefulness.

*H4*: PEOU is indirectly associated with a consumer’s FinTech adoption intention through perceived usefulness.

### 2.3 Trust

Trust is a central relational belief that shapes consumer decision-making under uncertainty, and it becomes especially salient in FinTech since digital financial services require users to rely on technology and providers when outcomes cannot be fully verified at the time of use (Mayer et al., 1995; McKnight et al., 2002; Li et al., 2023; Han and Zhao, 2026). Psychologically, trust operates as a critical mechanism for cognitive load reduction. In high-vulnerability contexts, when users experience a state of “felt safety,” their affective apprehension decreases (improving PEOU) and their expectancy of positive outcomes increases (improving PU) (McKnight et al., 2002). In this study, trust is positioned as an extension to TAM because FinTech adoption decisions are not driven only by instrumental beliefs (usefulness/ease), but also by the confidence that the provider and system will behave reliably and safeguard the consumer (Gefen et al., 2003).

Trust is generally defined as the readiness to accept vulnerability, contingent upon favorable expectations of another’s conduct and specific perceptions of their competence, goodwill, and honesty (Mayer et al., 1995; Zhao et al., 2024). In digital environments, trust is frequently treated as multidimensional—spanning institution-based trust, disposition to trust, trusting beliefs, and trusting intentions—which helps explain why it predicts behavior even when direct monitoring is limited (McKnight et al., 2002).

In technology-mediated exchanges, research integrating trust with TAM shows that behavioral intentions depend jointly on perceptions of the IT artifact (PU/PEOU) and trust in the vendor/service context, indicating that trust is not peripheral but comparable in importance to core TAM beliefs (Gefen et al., 2003). In financial-service channels specifically, initial trust is frequently emphasized as a decisive factor because consumers perceive heightened risk and potential loss, and empirical evidence in mobile banking shows that initial trust is a critical determinant in fostering the intention to adopt the service (Kim et al., 2009).

The central mediating function of trust has been repeatedly confirmed in recent literature regarding high-risk digital environments. Employing a stimulus-organism-response framework, Alamoudi et al. (2025) established that consumer trust acts as an indispensable mediator that translates system expectations into active digital payment adoption. Furthermore, Loke et al. (2026) demonstrated that trust significantly bridges the gap between performance expectancy, social influence, and adoption intentions—a process that is further amplified when backed by supportive government and regulatory interventions.

Trust can strengthen perceived usefulness as trusting beliefs imply positive expectations that the service will perform reliably and deliver the anticipated benefits, making consumers more likely to evaluate FinTech as instrumentally valuable (Mayer et al., 1995; McKnight et al., 2002). Trust also enhance perceived ease of use because confidence in the system/provider reduces uncertainty during interaction and supports more favorable user judgments about the effort required to complete transactions and navigate the service (McKnight et al., 2002; Gefen et al., 2003). Finally, trust may directly increase FinTech adoption intention because it lowers perceived vulnerability in digital financial exchange, and prior evidence shows that initial trust is vital for intention formation in mobile banking contexts (Gefen et al., 2003; Kim et al., 2009). Hence, the following hypotheses are posited:

*H5a*: Trust is positively associated with PU.

*H5b*: Trust is positively associated with PEOU.

*H5c*: Trust is positively associated with FinTech adoption intention.

### 2.4 Technology readiness

TR refers to consumers’ tendency to adopt and use new technologies to achieve objectives in their personal and professional lives (Parasuraman, 2000). Technology readiness index (TRI) conceptualizes TR through two motivating factors (optimism and innovativeness) and two inhibiting factors (discomfort and insecurity), capturing why some consumers welcome new technology while others resist it (Parasuraman, 2000; Parasuraman and Colby, 2015). TRI 2.0 further refines this measurement approach and supports its use as a stable, individual-difference lens for studying adoption of emerging digital services (Parasuraman and Colby, 2015).

Empirical work in service and digital contexts shows that TR meaningfully shapes consumers’ evaluations and adoption of self-service technologies, supporting its relevance for FinTech (which is largely self-service and app-mediated). TR has been used to explain how customers evaluate and adopt self-service technologies, indicating that readiness-related traits influence both attitudes and usage behavior toward technology-based service delivery channels (Liljander et al., 2006). Importantly for the present model, TR has also been integrated with TAM in prior research, demonstrating that TRI dimensions can act as antecedents to TAM beliefs (PU and PEOU), thereby providing a theoretically consistent pathway from personality-level readiness to intention-level acceptance (Lin and Hsieh, 2007; Walczuch et al., 2007).

The TR–trust linkage is theoretically defensible in FinTech because digital financial services involve perceived vulnerability and reliance on the system/provider, and TRI’s inhibitor dimensions explicitly reflect skepticism and lack of control that should reduce trust perceptions (Parasuraman and Colby, 2015). Likewise, motivator dimensions (optimism/innovativeness) should foster more favorable technology beliefs and greater willingness to engage with novel digital services, which is consistent with evidence that TR affects customers’ adoption of technology-based service channels and can translate into intention formation (Liljander et al., 2006; Lin and Hsieh, 2007). In TR–TAM integrations, the practical implication is that readiness traits shape how consumers cognitively appraise a system’s effort requirements (PEOU) and performance gains (PU), which then support adoption intention—an especially relevant mechanism for FinTech given its reliance on frequent, self-directed interactions.

Individuals with high TR (high optimism/innovativeness) tend to view technology as more useful, easier to use, and trustworthy, whereas low-TR individuals (high discomfort/insecurity) perceive higher risks and complexity (Lin and Hsieh, 2007). In FinTech contexts, these traits are critical because financial technologies often involve novel interfaces and intangible processes where user confidence is paramount.

Optimism denotes a favorable view of technology and the belief that it enhances control, flexibility, and efficiency (Parasuraman, 2000). Optimistic consumers tend to exhibit higher trust in technology providers because they focus on beneficial outcomes rather than potential failures (Parasuraman and Colby, 2015). Similarly, their favorable outlook leads them to perceive systems as more useful and easier to handle, and to exhibit stronger direct intention to adopt (Lin and Hsieh, 2007). In FinTech context, optimistic users are less likely to focus on negative outcomes, leading to higher levels of trust in the service provider (Walczuch et al., 2007). Furthermore, research confirms that optimism significantly reduces the perceived effort of using new systems (enhancing PEOU) and heightens the perception of functional benefits (enhancing PU) because optimistic users are more willing to cope with temporary technical difficulties (Walczuch et al., 2007; Godoe and Johansen, 2012). Consequently, optimism acts as a strong direct and indirect driver of adoption intention (Lin and Hsieh, 2007). Therefore, the following hypothesis is posited:

*H6a*: Optimism is positively associated with trust.

*H6b*: Optimism is positively associated with PU.

*H6c*: Optimism is positively associated with PEOU.

*H6d*: Optimism is positively associated with FinTech adoption intention.

Innovativeness is the tendency to be a pioneer and thought leader in adopting new technologies (Parasuraman, 2000). Innovative consumers possess higher intrinsic motivation and technical self-efficacy, which makes them more confident in their ability to master new FinTech interfaces, thereby increasing trust in the system’s reliability (Roy et al., 2018). Innovative users are intrinsically motivated to try new services, leading to higher initial trust in novel FinTech solutions (Parasuraman and Colby, 2015). Their technical curiosity makes them more skilled at navigating interfaces (higher PEOU) and more appreciative of functional benefits (higher PU), directly driving adoption intention (Lin and Hsieh, 2007). Studies show that highly innovative individuals perceive complex technologies as easier to use (higher PEOU) and are quicker to recognize novel utility (higher PU) compared to late adopters (Walczuch et al., 2007; Blut and Wang, 2020). This trait serves as a critical antecedent for both cognitive beliefs and direct behavioral intention. Therefore, the following hypothesis is posited:

*H7a*: Innovativeness is positively associated with trust.

*H7b*: Innovativeness is positively associated with PU.

*H7c*: Innovativeness is positively associated with PEOU.

*H7d*: Innovativeness is positively associated with FinTech adoption intention.

Discomfort represents a perceived lack of control and a sense of being overwhelmed by technology (Parasuraman, 2000). High discomfort reduces trust because users feel vulnerable and unsure about system reliability (Parasuraman and Colby, 2015). It also negatively impacts PEOU (due to anxiety) and PU (due to skepticism about benefits), thereby suppressing adoption intention (Lin and Hsieh, 2007). In financial contexts, this trait manifests as anxiety regarding the use of digital tools, which directly undermines trust by fostering skepticism about the system’s predictability (Parasuraman and Colby, 2015). Empirical research indicates that discomfort significantly lowers PEOU because anxious users perceive interactions as more effortful and complex (Walczuch et al., 2007). Similarly, it negatively impacts PU, as users preoccupied with the difficulty of the process fail to appreciate the system’s output benefits, ultimately suppressing adoption intention (Lin and Hsieh, 2007). Hence, the following hypothesis is posited:

*H8a*: Discomfort is negatively associated with trust.

*H8b*: Discomfort is negatively associated with PU.

*H8c*: Discomfort is negatively associated with PEOU.

*H8d*: Discomfort is negatively associated with FinTech adoption intention.

Insecurity involves skepticism about technology’s ability to work properly and concerns over potential negative consequences, such as data loss or fraud (Parasuraman, 2000). Insecurity directly undermines trust in the platform’s safety (Parasuraman and Colby, 2015). It also leads users to undervalue utility (lower PU) and perceive higher complexity (lower PEOU), ultimately inhibiting adoption intention (Lin and Hsieh, 2007). This inhibitor is particularly salient in FinTech, where it acts as a primary barrier to trust, as insecure users demand higher assurances of safety (Blut and Wang, 2020). Studies in e-services have found that insecurity leads to lower perceptions of utility (PU) and usability (PEOU) because the fear of risk overshadows potential gains and magnifies the perceived complexity of the transaction (Walczuch et al., 2007; Roy et al., 2018). Hence, the following hypothesis is posited:

*H9a*: Insecurity is negatively associated with trust.

*H9b*: Insecurity is negatively associated with PU.

*H9c*: Insecurity is negatively associated with PEOU.

*H9d*: Insecurity is negatively associated with FinTech adoption intention.

### 2.5 Conceptual framework

**FIGURE 1:**
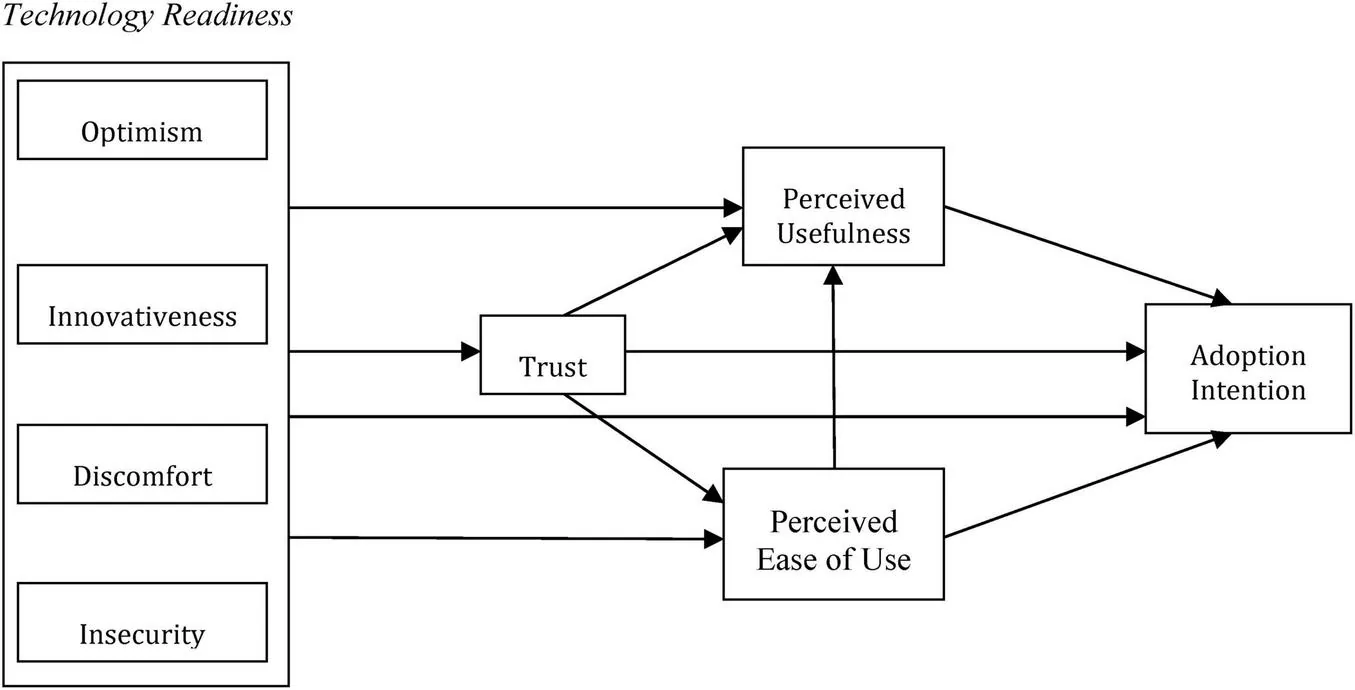
Conceptual framework.

Figure 1 presents the proposed conceptual framework, displaying the interconnections between the key constructs of the TAM—PU and PEOU—as well as the extended constructs of technology readiness and trust. Together, these elements illustrate their combined effect on FinTech adoption intention, emphasizing how technology readiness and trust enhance the traditional TAM framework to better explain users’ intention to adopt FinTech.

## 3 Methodology

### 3.1 Data

The target population comprised consumers in China’s financial services sector. Data were gathered in 2025 via an online questionnaire using a non-probability convenience sampling method. The survey link and QR code were disseminated through several Chinese social media platforms. The final dataset consisted of 336 valid responses. This sample size exceeds commonly recommended minimum requirements for PLS-SEM analysis. Following the widely adopted 10-times rule, the minimum sample size should be at least ten times the largest number of structural paths directed at any endogenous construct. Because the maximum number of incoming structural paths in the proposed model is seven, the recommended minimum sample size is 70 observations. The final sample of 336 responses substantially exceeds this requirement, providing adequate statistical power and supporting the robustness and stability of the structural model estimation (Kock and Hadaya, 2018; Hair et al., 2019).

As summarized in Table 1, the sample included 121 males (36.0%) and 215 females (64.0%). In terms of age, the largest group comprised participants aged 26–35 (173 respondents, 51.5%), followed by those aged 36–45 (105 respondents, 31.2%), 18–25 (42 respondents, 12.5%), and 46 and above (16 respondents, 4.8%). Most respondents (259 participants, 77.1%) reported holding a bachelor’s degree as their highest educational qualification. Additional demographic details are presented in Table 1.

**TABLE 1:** Participants (*N* = 336).

Variables	Frequency (%)
Gender	
Male	121 (36.0%)
Female	215 (64.0%)
Age	
18–25	42 (12.5%)
26–35	173 (51.5%)
36–45	105 (31.2%)
46 and above	16 (4.8%)
Qualification	
Senior high school and below	6 (1.8%)
Diploma	47 (14.0%)
Bachelor	259 (77.1%)
Master and above	24 (7.1%)

### 3.2 Instrument

The questionnaire consisted of eight constructs encompassing 24 items, all adapted from prior studies (see Table 2). Items were measured from 1 (“Strongly Disagree”) to 7 (“Strongly Agree”). Before the main survey, a two-stage pilot study was conducted to improve the quality of the measurement instrument. First, the questionnaire was administered to 10 university students from a nearby institution to evaluate the clarity of the instructions, question wording, and response format. Based on their feedback, several minor language revisions were made to improve readability while preserving the conceptual meaning of the original measurement items. Second, the revised questionnaire was administered to 30 randomly selected consumers at a shopping center to conduct a preliminary assessment of the measurement instrument. Construct reliability was evaluated using Cronbach’s alpha coefficients, which ranged from 0.77 to 0.91 across all constructs, indicating satisfactory internal consistency. The pilot study confirmed that the questionnaire possessed adequate clarity and reliability and was therefore suitable for the main survey. Three representative items were retained per construct to reduce respondent burden while preserving adequate construct coverage; the selected items capture the core conceptual domain of each construct and were drawn directly from the original validated scales and represent the core conceptual domain of each construct. All constructs demonstrated satisfactory reliability and validity (Cronbach’s alpha 0.798–0.921, CR 0.881–0.948, AVE > 0.50), confirming that the abbreviated scales adequately represent their underlying constructs.

**TABLE 2:** Measurement item sources.

Constructs	Number of items	Source
Optimism	3	(Parasuraman and Colby, 2015)
Innovativeness	3	(Parasuraman and Colby, 2015)
Discomfort	3	(Parasuraman and Colby, 2015)
Insecurity	3	(Parasuraman and Colby, 2015)
Trust	3	(Kim et al., 2009)
Perceived usefulness	3	(Davis et al., 1989)
Perceived ease of use	3	(Davis et al., 1989)
Adoption intention	3	(Davis et al., 1989)

The complete questionnaire with all 24 items is provided in Appendix 1.

### 3.3 Analytical approaches

The analysis followed a two-step approach, evaluating the measurement model and then the structural model (Hair et al., 2020). First, the reliability and validity of the measurement instruments were assessed. Next, the hypothesized research model was examined using PLS-SEM.

## 4 Analysis and results

### 4.1 Common method bias

Since common method bias (CMB) can compromise the findings’ validity, the study employed both procedural and statistical remedies to reduce and evaluate potential bias. Procedurally, respondent anonymity was ensured, clear instructions were provided, and the questionnaire was kept concise to minimize fatigue and confusion, avoiding complex or ambiguous items (Weijters and Baumgartner, 2012). Given that the survey was administered in China, the questionnaire was used back-translation method to confirm conceptual equivalence of the Chinese and English version.

Statistically, two diagnostic tests were performed to evaluate the presence of CMB. First, Harman’s single-factor test was employed (Podsakoff et al., 2003). A single factor accounting for more than 50% of the total variance indicates potential bias (Harman, 1976). In this study, the highest variance explained by a single factor was 44.487%, which is below the critical threshold, suggesting no significant CMB issue. Second, collinearity statistics were examined following the guidelines proposed by (Kock, 2015). Variance Inflation Factor (VIF) values exceeding 3.3 indicate problematic collinearity and may signal the presence of CMB. In this analysis, all VIF values ranged between 1.129 and 2.754, further confirming the absence of CMB in the dataset.

### 4.2 Measurement model assessment

Internal consistency and construct reliability were performed to test the measurement model’s reliability (Hair et al., 2010). Internal consistency was measured using Cronbach’s α coefficients, with a minimum acceptable value of 0.6 (Nunnally, 1978). As presented in Table 3, Cronbach’s α values ranged from 0.798 to 0.921, exceeding the recommended threshold. In addition, construct reliability was examined using Composite Reliability (CR), for which (Hair et al., 2010) recommend a threshold of 0.7. The CR values for all constructs ranged between 0.881 and 0.948. These results collectively indicate a high level of internal consistency and measurement reliability across the constructs.

**TABLE 3:** Reliability and validity measures.

				Discriminant validity: Fornell-Larcker criterion							
Construct	Cronbach’s *a*	CR	AVE	Intent	Discom	Inn	Insec	Opt	PEOU	PU	Trust
Intent	0.894	0.934	0.826	0.909	0.927	0.915	0.916	0.848	0.887	0.844	0.906
Discom	0.921	0.948	0.858	−0.123							
Inno	0.903	0.939	0.838	0.744	−0.142						
Insec	0.906	0.940	0.839	−0.197	0.634	−0.189					
Opt	0.805	0.885	0.720	0.704	−0.089	0.702	−0.224				
PEOU	0.864	0.917	0.786	0.688	−0.171	0.636	−0.207	0.682			
PU	0.798	0.881	0.712	0.680	−0.091	0.591	−0.202	0.703	0.712		
Trust	0.891	0.932	0.821	0.690	−0.095	0.649	−0.278	0.751	0.647	0.648	

Intent, adoption intention; Discom, discomfort; Inno, innovativeness; Insec, Insecurity; Opt, optimism;PEOU, perceived ease of use; PU, perceived usefulness.

To assess validity, two dimensions were examined: convergent validity and discriminant validity. Convergent validity was examined using the Average Variance Extracted (AVE) and outer loadings derived from Confirmatory Factor Analysis (CFA), reflecting the extent to which multiple indicators of the same construct are positively correlated (Hair et al., 2010). Adequate convergent validity is established when item outer loadings are greater than 0.60 and AVE values exceed 0.50 (Hair et al., 2010). As shown in Tables 3, 4, the AVE values and outer loadings confirmed that the model achieved satisfactory convergent validity.

**TABLE 4:** Scale properties of the measurement model.

Construct	Item	CFA loading	T-statistic
Discomfort	Discom1	0.878	14.938
	Discom 2	0.956	30.178
	Discom 3	0.943	28.578
Innovativeness	Inno1	0.904	77.417
	Inno2	0.930	111.617
	Inno3	0.911	87.273
Insecurity	Insec1	0.883	39.070
	Insec 2	0.930	82.223
	Insec 3	0.934	87.140
Adoption intention	Intent1	0.917	89.015
	Intent2	0.902	70.611
	Intent3	0.907	71.307
Optimism	Opt1	0.863	54.177
	Opt2	0.831	42.835
	Opt3	0.850	41.625
PEOU	PEOU1	0.880	57.594
	PEOU2	0.889	62.875
	PEOU3	0.891	64.705
PU	PU1	0.862	49.154
	PU2	0.844	43.206
	PU3	0.825	33.899
Trust	Trust1	0.911	72.202
	Trust2	0.924	94.690
	Trust3	0.882	57.131

Intent, adoption intention; Discom, discomfort; Inno, innovativeness; Insec, Insecurity; Opt, optimism; PEOU, perceived ease of use; PU, perceived usefulness.

Discriminant validity examines whether constructs are distinct from one another and indicate different conceptual dimensions (Siekpe, 2005) The Fornell–Larcker criterion was used to assess discriminant validity, which is achieved when the square root of each construct’s AVE is greater than its strongest correlation with any other construct (Fornell and Larcker, 1981). Discriminant validity was further evaluated using the heterotrait-monotrait ratio of correlations (HTMT), which is recommended as a more sensitive criterion for assessing discriminant validity in PLS-SEM (Henseler et al., 2015). All HTMT values were below the recommended threshold of 0.90 (maximum = 0.87). In addition, the upper bound of the bootstrapped 95% confidence interval for every construct pair did not include 1.00, providing further evidence that discriminant validity was satisfactorily established (Franke and Sarstedt, 2019). The AVE statistics (Table 3) and outer loadings (Table 4) show adequate convergent validity of the measurement model.

### 4.3 Structural model assessment

The structural model results show that TAM variables (PU and PEOU), together with trust and selected technology readiness dimensions, significantly explain consumers’ FinTech adoption intention. Hypothesis testing results (path coefficients and *t*-values) are reported in Table 5, and the model explains substantial variance in the endogenous constructs, with R^2^ values of 0.610 (Trust), 0.606 (PU), 0.546 (PEOU), and 0.682 (Adoption intention). Using common PLS-SEM guidelines (0.67/0.33/0.19 as substantial/moderate/weak) (Chin, 1998), the R^2^ values indicate at least moderate explanatory power for all endogenous variables, particularly the substantial explanatory power for adoption intention.

**TABLE 5:** Path assessment.

Hypothesis relationship	Path coefficient	*t*-values	Decision
H1: PU → AI	0.183**	2.770	Accepted
H2: PEOU → AI	0.157*	2.269	Accepted
H3: PEOU → PU	0.394*	5.571	Accepted
H4: PEOU → PU → AI	0.072**	2.639	Accepted
H5a: Trust → PU	0.136*	2.005	Accepted
H5b: Trust → PEOU	0.251**	2.936	Accepted
H5c: Trust → AI	0.172**	2.561	Accepted
H6a: Optimism → Trust	0.549**	10.966	Accepted
H6b: Optimism → PU	0.298**	3.844	Accepted
H6c: Optimism → PEOU	0.332**	4.386	Accepted
H6d: Optimism → AI	0.085	1.330	Declined
H7a: Innovativeness → Trust	0.245**	4.326	Accepted
H7b: Innovativeness → PU	0.042	0.652	Declined
H7c: Innovativeness → PEOU	0.233**	3.213	Accepted
H7d: Innovativeness → AI	0.366**	6.115	Accepted
H8a: Discomfort → Trust	0.095	1.906	Declined
H8b: Discomfort → PU	0.045	1.045	Declined
H8c: Discomfort → PEOU	−0.122*	2.223	Accepted
H8d: Discomfort → AI	−0.015	0.355	Declined
H9a: Insecurity → Trust	−0.169**	3.489	Accepted
H9b: Insecurity → PU	−0.037	0.808	Declined
H9c: Insecurity → PEOU	0.059	1.108	Declined
H9d: Insecurity → AI	0.019	0.473	Declined
Total effects			
Optimism → AI	0.356**	6.665	
Innovativeness → AI	0.489**	8.828	
Discomfort → AI	−0.010	0.229	
Insecurity →AI	−0.018	0.435	
Squared multiple correlation (R2)			
Trust	0.610	14.510	
PU	0.606	14.490	
PEOU	0.546	10.397	
Adoption intention	0.682	20.149	

**p < 0.01, *p < 0.05. PEOU, perceived ease of use; PU, perceived usefulness; AI, adoption intention.

Predictive relevance was evaluated via the Stone–Geisser *Q*^2^ obtained through blindfolding; a *Q*^2^ greater than zero signals that the model has predictive capability for the corresponding endogenous construct (Hair et al., 2019). The blindfolding procedure was conducted using an omission distance of 5. The *Q*^2^ values for adoption intention (0.547), perceived ease of use (0.416), perceived usefulness (0.416), and trust (0.493) were positive, indicating that the model possesses predictive relevance. These results suggest that the proposed model explains the relationships between constructs and demonstrates adequate predictive capabilities for endogenous variables.

#### 4.3.1 Direct effects on adoption intention

Adoption intention is positively influenced by PU (β = 0.183, *t* = 2.770) and PEOU (β = 0.157, *t* = 2.269), supporting H1 and H2. Trust also has a significant positive effect on adoption intention (β = 0.172, t = 2.561), supporting H5c. Among technology readiness dimensions, innovativeness has a significant direct positive effect on adoption intention (β = 0.366, *t* = 6.115), while optimism, discomfort, and insecurity show non-significant direct effects, leading to the rejection of H6d, H8d, and H9d.

#### 4.3.2 Antecedents of trust and TAM beliefs

Trust is significantly increased by optimism (β = 0.549, *t* = 10.966) and innovativeness (β = 0.245, *t* = 4.326), and significantly reduced by insecurity (β = −0.169, *t* = 3.489), supporting H6a, H7a, and H9a, while discomfort is not significant (H8a rejected). PU is positively predicted by PEOU (β = 0.394, *t* = 5.571), trust (β = 0.136, *t* = 2.005), and optimism (β = 0.298, *t* = 3.844), supporting H3, H5a, and H6b, whereas innovativeness, discomfort, and insecurity do not significantly affect PU (H7b, H8b, H9b rejected). PEOU is positively affected by trust (β = 0.251, *t* = 2.936), optimism (β = 0.332, *t* = 4.386), and innovativeness (β = 0.233, *t* = 3.213), but negatively affected by discomfort (β = −0.122, *t* = 2.223), supporting H5b, H6c, H7c, and H8c, while insecurity is not significant (H9c rejected).

#### 4.3.3 Total effects of technology readiness on adoption intention

The indirect effect of PEOU on adoption intention through PU is significant (β = 0.072, t = 2.639), supporting the mediation hypothesis (H4). Table 6 indicates that optimism has a non-significant direct effect on adoption intention (β = 0.085) but a significant total effect (β = 0.356) due to indirect paths, while innovativeness demonstrates both significant direct (β = 0.366) and total effects (β = 0.489). In contrast, discomfort and insecurity show non-significant total effects on adoption intention (β = −0.010 and β = −0.018, respectively), implying their influence does not translate into overall changes in adoption intention in this model.

**TABLE 6:** Effects of technology readiness on adoption intention.

Factor	Effect	Adoption intention	Significance level
Optimism	Direct effect	0.085	Insignificant
	Total indirect effects	0.270	**
	Total effect	0.356	**
Innovativeness	Direct effect	0.366	**
	Total indirect effects	0.123	**
	Total effect	0.489	**
Discomfort	Direct effect	−0.015	Insignificant
	Total indirect effects	0.005	Insignificant
	Total effect	−0.010	Insignificant
Insecurity	Direct effect	0.019	Insignificant
	Total indirect effects	−0.036	Insignificant
	Total effect	−0.018	Insignificant

**p ≤ 0.01, insignificant at *p* > 0.05.

## 5 Discussion

The structural model suggests a layered adoption process in which TAM beliefs (PU and PEOU) operate as the most proximal drivers of intention, trust both shapes these beliefs and contributes independently, and technology readiness mainly influences adoption indirectly by conditioning trust and TAM beliefs rather than acting as a uniform direct determinant (Davis, 1989; Parasuraman, 2000; Venkatesh and Davis, 2000; Gefen et al., 2003). The model shows strong explanatory power for adoption intention, suggesting that the integrated TAM–trust–technology readiness framework captures substantial variance in intention.

### 5.1 Main findings

Rather than simply demonstrating that Technology Readiness, trust, and TAM jointly predict FinTech adoption—a relationship that has been documented in previous technology acceptance studies (Lin and Hsieh, 2007; Walczuch et al., 2007; Blut and Wang, 2020)—the present findings reveal that these constructs are connected through differentiated psychological pathways. Specifically, the results indicate that the four technology readiness dimensions do not operate as interchangeable antecedents. Instead, each readiness dimension selectively influences different psychological states before behavioral intention is formed. This finding extends the psychological interpretation of Technology Readiness by demonstrating that stable technological predispositions are translated into adoption intention through distinct cognitive and affective processes rather than through a single, uniform acceptance mechanism (Parasuraman, 2000; Parasuraman and Colby, 2015). Consequently, the proposed trait-to-state-to-intention mechanism should be understood as a psychological explanation of how enduring personality characteristics shape technology adoption under conditions of financial uncertainty.

The study indicates that consumers’ FinTech adoption intention is primarily governed by expected functional value (PU) and perceived interaction effort (PEOU), with the mediation of PEOU through PU reinforcing the classic TAM mechanism that “ease” matters partly because it increases perceived benefits (Davis, 1989; Venkatesh and Davis, 2000). A psychologically meaningful interpretation is that, in app-based finance, PEOU can also act as a quality cue (smooth interaction as a signal of competence), which can elevate usefulness judgments beyond pure effort reduction and thereby strengthen the PEOU → PU pathway. This result is consistent with TAM research across mobile banking (Zhou, 2011; Shaikh and Karjaluoto, 2015) and FinTech payments (Daragmeh et al., 2021), confirming the PU-PEOU-intention architecture generalizes to the Chinese FinTech context. However, the PEOU-PU path observed here is stronger than in early organizational TAM studies (Davis et al., 1989), suggesting that in app-based financial services, interface fluency serves dual functions: reducing instrumental effort and signaling competence, thereby amplifying usefulness judgments.

The findings further imply that FinTech adoption is not purely an instrumental decision; rather, trust both increases intention directly and improves PU and PEOU, which is consistent with trust-integrated acceptance models emphasizing vulnerability and uncertainty in digital exchange (McKnight et al., 2002; Gefen et al., 2003; Kim et al., 2009). Psychologically, trust can be read as a state of “felt safety/assurance” that makes consumers more willing to engage with the interface (higher PEOU as reduced apprehension) and more willing to believe promised benefits will materialize (higher PU as more favorable expectancy). At the same time, trust may partially capture broader institutional confidence in the provider category or the digital finance environment (not only the focal app), which could elevate intention even when consumers have not fully processed detailed utility attributes. This tripartite trust mechanism mirrors findings in e-commerce (Gefen et al., 2003) and mobile banking (Kim et al., 2009). However, the trust-PEOU path exceeds trust-PU, reversing the pattern in online shopping where trust-PU dominates (Gefen et al., 2003). This suggests that in FinTech, trust functions primarily as an anxiety-reduction mechanism lowering perceived interaction cost, rather than purely as a benefit-amplification mechanism.

Technology readiness operates as a trait-level lens that shapes consumers’ psychological states—trust, PU (cognitive appraisal of utility), and PEOU (experiential/affective sense of effort and comfort)—which then shape intention. This pattern supports TR–TAM integration logic (Lin and Hsieh, 2007; Walczuch et al., 2007) by showing that traits do not necessarily trigger intention directly; instead, they systematically bias how consumers interpret uncertainty (trust) and interaction demands (PEOU), and how they translate those experiences into perceived value (PU).

The differentiated effects across TRI dimensions indicate that readiness is not monolithic and should be treated as four distinct antecedents with different psychological channels (Parasuraman, 2000; Parasuraman and Colby, 2015; Blut and Wang, 2020). Optimism appears primarily as a belief-shaping disposition (via trust, PU, and PEOU) rather than a direct trigger of intention, implying that positive technology orientation becomes adoption-relevant only after it is “converted” into trust and favorable appraisals. Innovativeness functions more as a behavioral catalyst by directly predicting intention and improving PEOU (and trust) even without significantly increasing PU, suggesting that early adopters may act on exploration/self-efficacy motives rather than strictly utilitarian appraisal. Among the inhibitor dimensions, discomfort primarily manifests as a usability-related psychological barrier by reducing perceived ease of use, whereas insecurity mainly undermines users’ trust without directly altering perceived usefulness or perceived ease of use. These findings suggest that negative technology readiness traits do not generate a single generalized resistance to FinTech adoption but instead influence distinct psychological states through different mechanisms. This direct innovativeness-intention path, which bypasses the cognitive utility evaluation central to TAM, is theoretically consistent with TRI’s conceptualization of innovativeness as a propensity to pioneer new technologies (Parasuraman and Colby, 2015). Innovative consumers adopt FinTech through intrinsic exploration motives and technological self-efficacy—engaging with novel technology is intrinsically rewarding and identity-consistent, rather than contingent on a deliberative cost-benefit calculus. Meta-analytic evidence supports this interpretation, showing innovativeness exhibits stronger direct relationships with technology usage than other TR dimensions (Blut and Wang, 2020). The non-significant total effects of discomfort and insecurity on adoption intention should be interpreted within China’s highly digitalized financial ecosystem. Digital payment platforms such as WeChat Pay and Alipay have become essential infrastructure supporting everyday transactions. Consequently, even consumers experiencing discomfort or insecurity may continue using FinTech because participation in daily economic and social activities increasingly depends on these technologies. Under such conditions, inhibitor traits continue to shape users’ psychological evaluations but exert weaker direct effects on behavioral intention. This systemic dependence can attenuate the behavioral consequences of negative TR traits: inhibitors shape psychological states (trust, PEOU) but may not translate into reduced intention when environmental pressure to adopt is sufficiently strong. This identifies an important boundary condition for Technology Readiness Theory—in highly digitalized societies, the trait-intention link may be moderated by whether the technology constitutes infrastructural necessity rather than optional innovation.

Collectively, these findings suggest that Technology Readiness should be conceptualized as a differentiated psychological system rather than a single dispositional construct. Whereas previous TR–TAM studies have primarily examined whether technology readiness facilitates or inhibits technology acceptance (Lin and Hsieh, 2007; Walczuch et al., 2007), the present results demonstrate that different readiness dimensions activate different psychological routes. Optimism primarily reshapes users’ cognitive evaluations through trust, perceived usefulness, and perceived ease of use, whereas innovativeness additionally exhibits an intrinsic motivational pathway directly influencing behavioral intention. In contrast, discomfort primarily manifests as interaction-related psychological resistance, whereas insecurity mainly undermines trust. This differentiated perspective extends Technology Readiness Theory by explaining how specific readiness dimensions operate through distinct psychological mechanisms rather than functioning as equivalent antecedents (Parasuraman and Colby, 2015; Blut and Wang, 2020).

The findings also suggest that the relationship between personality traits and behavioral intention depends on the societal role of the technology being adopted. When digital financial technologies become institutional infrastructure rather than optional innovations, environmental dependence may attenuate the behavioral consequences of individual psychological resistance. Consequently, the present findings identify an important contextual boundary condition for Technology Readiness Theory by suggesting that the relationship between readiness traits and adoption intention varies according to the degree to which technologies are socially and institutionally embedded (Parasuraman and Colby, 2015; Yang and Zhang, 2022).

### 5.2 Theoretical contributions

The study contributes to the literature on cyberpsychology and human-computer interaction in several ways. First, the study advances Technology Readiness research by demonstrating that the four readiness dimensions should not be interpreted as a homogeneous set of antecedents. Instead, optimism, innovativeness, discomfort, and insecurity exhibit distinct psychological functions that selectively influence trust, perceived usefulness, perceived ease of use, and behavioral intention. This differentiated pathway perspective extends previous TR–TAM integration studies (Lin and Hsieh, 2007; Walczuch et al., 2007; Blut and Wang, 2020) by explaining not only whether technology readiness influences adoption, but also how different readiness traits exert their effects through distinct psychological mechanisms (Parasuraman and Colby, 2015).

Second, the study refines trust-integrated TAM by positioning trust as an upstream psychological state that simultaneously shapes both cognitive evaluations and behavioral intention. Rather than functioning merely as an additional predictor of technology acceptance (Gefen et al., 2003; Kim et al., 2009), trust operates as a mechanism through which users interpret uncertainty before forming judgments regarding usefulness and ease of use. This clarifies the psychological sequence through which financial uncertainty influences technology acceptance.

Third, the findings provide empirical support for a differentiated trait-to-state-to-intention mechanism linking stable personality characteristics with transient cognitive and affective evaluations. Consistent with psychological perspectives that distinguish enduring dispositions from situational cognitive states (Parasuraman, 2000; Lin and Hsieh, 2007), the results demonstrate that technology acceptance emerges through multiple psychological routes rather than a single generalized antecedent–belief–intention process. This refines existing acceptance theory by providing a more psychologically explicit explanation of how individual differences shape technology adoption.

Finally, the study identifies a FinTech-specific contextual boundary condition for Technology Readiness Theory. The findings suggest that when digital financial platforms become embedded as essential societal infrastructure, environmental dependence may weaken the direct behavioral influence of negative readiness traits such as discomfort and insecurity, even though these traits continue to shape users’ psychological evaluations. This extends Technology Readiness Theory beyond optional technology contexts and highlights the importance of institutional environments in understanding technology adoption (Parasuraman and Colby, 2015; Yang and Zhang, 2022).

### 5.3 Applied psychological implications

The findings provide actionable insights for the design of digital financial systems, emphasizing cognitive ergonomics and psychological intervention over traditional marketing strategy. First, because adoption intention is deeply rooted in cognitive appraisals and psychological safety, system design must prioritize transparent signaling. Trust operates as an upstream enabler of usefulness and ease of use (McKnight et al., 2002; Gefen et al., 2003); therefore, trust-building mechanisms—such as visible security cues, transparent data practices, and clear recourse options—must be embedded directly into the user interface. Alleviating insecurity at the point of interaction prevents skepticism from undermining the user’s perception of the system’s utility (Parasuraman and Colby, 2015).

Second, the differentiated pathways of technology readiness suggest that digital platforms should employ adaptive interface designs that cater to varying psychological profiles (Parasuraman, 2000). For user segments exhibiting high discomfort and insecurity, platforms should provide cognitive scaffolding, such as guided onboarding, “safe-to-try” sandbox environments, and immediate access to human support. These interventions directly target the affective friction that reduces PEOU and trust (Walczuch et al., 2007). Conversely, for users driven by innovativeness and optimism, interfaces can allow for more exploratory learning and early access to advanced features, leveraging their intrinsic technical self-efficacy and higher tolerance for ambiguity (Lin and Hsieh, 2007).

Beyond interface optimization, the findings have broader implications for multiple stakeholders within the FinTech ecosystem. For financial institutions, trust-building should be regarded as a strategic priority through transparent communication, robust cybersecurity measures, privacy protection, and responsive customer support. Furthermore, implementation strategies should recognize that consumers differ in their levels of technology readiness, requiring tailored onboarding and support for users experiencing discomfort or insecurity. For policymakers and regulators, the findings underscore the importance of effective regulatory frameworks that enhance institutional trust through strong consumer protection, cybersecurity governance, and transparent regulatory oversight. Finally, improving digital financial literacy represents an important complementary strategy. Educational initiatives that strengthen consumers’ understanding of digital financial services, cybersecurity, and fraud prevention can reduce psychological barriers, enhance trust, and facilitate the responsible adoption of FinTech services.

## 6 Conclusion, implication, and limitations

### 6.1 Conclusion

The study set out to extend the Technology Acceptance Model by incorporating trust and the four dimensions of technology readiness to explain consumers’ intention to adopt FinTech services in China. Using PLS-SEM, the model demonstrated substantial explanatory power for adoption intention, indicating that the combination of perceived usefulness, perceived ease of use, trust, and selected technology readiness dimensions offers a robust account of why consumers intend to adopt FinTech. The pattern of supported and rejected hypotheses suggests that adoption is best understood as a multistage process in which technology beliefs are proximal drivers, trust operates both directly and indirectly through those beliefs, and technology readiness primarily shapes upstream belief formation rather than uniformly exerting direct effects on intention.

### 6.2 Limitations and recommendations

Several limitations are observed. First, because the study employed a cross-sectional survey using self-reported data, the estimated structural relationships should be interpreted as theoretically supported associations rather than definitive causal effects. Although procedural and statistical assessments indicated that common method bias was unlikely to threaten the validity of the findings, residual method variance cannot be completely excluded. Future research should employ longitudinal, experimental, or multi-source designs to provide stronger evidence regarding causal relationships. Second, the study employed convenience sampling through online social media platforms within China, which may limit the representativeness of the sample and the generalizability of the findings to other cultural, institutional, and regulatory environments. Future studies should employ probability sampling where feasible and conduct cross-national comparisons to examine whether the proposed psychological mechanisms remain stable across different FinTech ecosystems. Finally, the study focuses on intention which intention–behavior gap may exist for FinTech adoption and whether factors such as switching costs condition the translation of intention into actual switching/adoption behavior need be addressed (Zhao, 2025).

## Data Availability

The raw data supporting the conclusions of this article will be made available by the authors, without undue reservation.

## Appendix

Appendix 1 | Questionnaire Items.

All items were measured on a 7-point Likert scale (1 = Strongly Disagree, 7 = Strongly Agree). Items were adapted from validated scales as indicated in Table 2.

*Perceived usefulness (PU)*

PU1: Using FinTech services improves my banking efficiency.

PU2: FinTech enables me to accomplish financial tasks more quickly.

PU3: FinTech services enhance the effectiveness of my banking activities.

*Perceived ease of use (PEOU)*

PEOU1: Learning to use FinTech services is easy for me.

PEOU2: Interacting with FinTech platforms is clear and understandable.

PEOU3: I find FinTech systems easy to use.

*Trust*

Trust1: I believe FinTech services are trustworthy.

Trust2: I believe FinTech providers keep my best interests in mind.

Trust3: I believe FinTech platforms are reliable and dependable.

*Optimism*

Opt1: Technology gives me more control over my daily financial life.

Opt2: FinTech services make banking more convenient for me.

Opt3: I believe technology increases the quality of my financial decisions.

*Innovativeness*

Inno1: I am among the first in my social group to try new financial technologies.

Inno2: I like experimenting with new FinTech applications.

Inno3: I enjoy exploring innovative digital financial solutions.

*Discomfort*

Discom1: I feel overwhelmed when there are too many FinTech options.

Discom2: It is difficult for me to understand complex FinTech features.

Discom3: I feel uncomfortable using FinTech without assistance.

*Insecurity*

Insec1: I doubt that FinTech systems work properly all the time.

Insec2: I hesitate to rely on FinTech for important financial transactions.

Insec3: I fear that FinTech systems may fail when I need them most.

*Adoption intention*

Intent1: I intend to use FinTech services regularly.

Intent2: I will use FinTech services whenever possible.

Intent3: I plan to use FinTech services in the future.
